# THE COMBINED USE OF NON-INVASIVE NEUROSTIMULATION WITH INTERACTIVE DIGITAL TECHNOLOGIES IN SPINAL CORD INJURY REHABILITATION: A SCOPING REVIEW

**DOI:** 10.2340/jrm.v58.45747

**Published:** 2026-07-21

**Authors:** Samuel David WILLIAMSON, Magnus Møller ANDERSEN, Anders Orup AABY, Marianne Rahbek VESTERGAARD, Sophie Lykkegaard RAVN

**Affiliations:** 1 Specialized Hospital for Polio and Accident Victims, Rødovre; 2 Department of Psychology, University of Southern Denmark, Denmark

**Keywords:** electric stimulation, electric stimulation therapy, serious games, recovery of function, virtual reality

## Abstract

**Objective:**

To map the empirical evidence regarding the combined use of non-invasive neurostimulation with interactive digital technologies in spinal cord injury rehabilitation.

**Design:**

Scoping review.

**Methods:**

Seven databases facilitated a systematic, 3-block search. Additional searches were performed in Google Scholar, PEDro, and via forward and backward snowballing. Studies were required to report original, clinical data from individuals with spinal cord injury participating in interventions that combine non-invasive neurostimulation with interactive digital technologies. Screening and data extraction were performed by 2 independent reviewers according to pre-specified criteria.

**Results:**

A total of 494 unique records were identified via the database searches, in addition to the 34 unique records identified via additional searches. Twelve studies met the eligibility criteria. Eleven studies delivered peripheral neurostimulation (i.e., neuromuscular/functional electrical stimulation), and 1 study delivered spinal-directed neurostimulation (i.e., transcutaneous spinal cord stimulation). Interactive digital technologies ranged from manual interfaces controlling 2D avatars to immersive, autonomous closed-loop feedback systems.

**Conclusion:**

The accumulated evidence suggests that combining non-invasive neurostimulation with interactive digital technologies may enhance task-specific motor recovery and engagement in spinal cord injury rehabilitation. However, the research area is characterized by a low evidentiary standard, prompting a need for higher-quality studies to establish long-term clinical viability.

## LAY ABSTRACT

This review looked at how combining electrical stimulation with interactive digital tools, such as virtual reality or video games, may support rehabilitation after spinal cord injury. We found 12 studies involving 51 participants. These approaches often used electrical stimulation to activate muscles while people interacted with digital environments, for example by cycling, balancing, or playing game-like tasks. Overall, studies reported improvements in movement, balance, hand function, and independence. Many also reported that the training was enjoyable and motivating, which may help people stay engaged in rehabilitation. However, most studies were small and early-stage, meaning the results are not yet strong enough to draw firm conclusions. More high-quality research is needed to understand whether these combined approaches lead to lasting benefits and can be widely used in clinical practice.

Spinal cord injury (SCI) is associated with profound sensorimotor impairments (1) and autonomic dysfunction (2), thereby necessitating interventions that promote neuroplasticity and functional recovery. For example, non-invasive neurostimulation techniques are widely and traditionally administered to modulate neuromuscular or neural function in this population. Techniques such as functional electrical stimulation (FES) and transcutaneous spinal cord stimulation (tSCS) are reported to promote fine motor control (3, 4) and lower limb mobility (5, 6), in addition to improving autonomic functions such as bladder and bowel control (7, 8) in SCI rehabilitation. However, to achieve results, these techniques are often required to be delivered consistently, at high volume, and with sufficient intensity over a sustained period (9, 10). As functional improvements are also contingent on task-specific practice and user engagement (11), integration with existing and developing technologies has been proposed as a means to motivate participants, to maintain adherence to rehabilitation protocols (12), and to provide multimodal and additive benefits (13, 14). As such, interactive digital technologies, ranging from 2D smartphone and laptop screens to immersive 3D virtual reality (VR) displays, have become established as viable tools in neurological rehabilitation. For example, these technologies can provide real-time, adaptable biofeedback (15), simulate immersive, natural environments relevant to task-specific training (16), and present interactive exergame challenges encouraging desired movements or objectives (17).

The combined use of non-invasive neurostimulation and interactive digital technologies appears well established, particularly within the stroke literature, where systematic reviews and meta-analyses have reported promising findings across upper-extremity and motor domains. For example, a recent systematic review of VR combined with FES for upper-extremity rehabilitation (18) identified 4 further interventions in the post-stroke population, and 9 studies were included in a separate meta-analysis of BCI interventions in post-stroke motor rehabilitation (19). Evidence for the combined use of interactive digital technologies and non-invasive neurostimulation is also mapped across the wider neurological literature. For instance, 13 articles, excluding interventions performed in healthy populations, were included in a recent systematic review of VR combined with non-invasive brain stimulation across diverse rehabilitation contexts (20). Here, interventions relevant to the current review were identified in stroke, cerebral palsy, and multiple sclerosis participants, underlining the growing body of research in this field across different populations. By comparison, the SCI literature remains sparse, which emphasizes the importance of mapping and advancing this line of research within the target population.

Currently, no scoping review has systematically mapped the available, empirical evidence in the SCI population. This was the aim of the present study. More specifically, this review investigated how the synchronous combination of non-invasive neurostimulation and interactive digital technologies was operationalized in studies in SCI rehabilitation settings, including how the combination was achieved, for which purposes, and what outcomes were reported. This work was intended to inform future research and clinical applications in integrative SCI rehabilitation.

## METHODS

The present review was reported in accordance with the Preferred Reporting Items for Systematic reviews and Meta-Analyses extension for Scoping Reviews (PRISMA-ScR) (21) (see Fig. S1). The associated protocol was registered in advance with the Open Science Framework on 8 January 2026 (https://doi.org/10.17605/OSF.IO/A7QHR).

### Information sources and search strategy

Three search blocks containing synonyms or terms associated with (*i*) SCI, (*ii*) interactive digital technology, and (*iii*) stimulation were developed, piloted, and refined in 7 databases, namely, Embase (via Ovid), MEDLINE (via Ovid), APA PsycInfo (via Ovid), Web of Science, Scopus, CINAHL (via EBSCOhost), and the Cochrane Central Register of Controlled Trials (CENTRAL). Databases were originally searched from inception to their most recent update as of 6 January 2026 and updated on 26 May 2026. Search terms were based on a comprehensive reading of relevant literature and influenced by several existing search strategies within the subject area. No filters were used to limit the search. Subject headings were adapted for each database’s unique indexing system. Where new subject headings were introduced to the string, corresponding free-text search terms were retroactively included. Therefore, a uniform string of free-text search terms was used across all databases at the title, abstract, and keyword level or similar. Boolean operators were used to combine search terms within (“OR”) and across (“AND”) blocks. This procedure was carried out multiple times to guarantee uniformity prior to conducting the final searches on 6 January 2026, updated on 26 May 2026. The final search string is available as Fig. S2.

### Additional searches

Two types of additional searches were carried out by the second author under supervision from the first author. On 15 January 2026, 5 unique combinations of the search terms were entered into Google Scholar (22) to supplement the original database searches. For pragmatic reasons and based on the known diminishing returns associated with screening progressively lower-ranked Google Scholar records, the first 50 results attributed to each combination, having first been sorted by relevance, were screened against the eligibility criteria. On 15 January 2026, the search term “spinal cord injury” was used to browse PEDRO’s archive of clinical trials (23). Next, all included studies and tagged records (see description of procedure below) were subject to forward snowballing, performed via Google Scholar, and backward snowballing, performed manually. All studies considered potentially relevant by the second author were imported into Covidence and were independently double-screened by 2 authors based on the same eligibility criteria as described below at the full-text level. Finally, after completing the full screening process, the second author conducted searches on Google to verify that no information from all included studies was corrected or retracted post-publication.

### Eligibility criteria

Inclusion was dependent on studies being published in English, Danish, Norwegian, or Swedish, reflecting the languages the author group can reliably interpret without external assistance. Studies had to be conducted among individuals living with SCI and consist of original, empirical, and clinical data published in either peer-reviewed research articles, conference abstracts or posters, dissertations, or theses. In cases of relevant dual publications, defined as extensions of the same research reported and disseminated in different formats, only the version judged to provide the most complete and usable information for data extraction was included. This decision was made to avoid double or triple reporting, and subsequent data distortion. Relevant sister publications were excluded under a category labelled “dual publication”. Additionally, studies had to have been performed in a rehabilitative rather than a recreational context and were required to evaluate a neurostimulation approach targeting the sensory or motor pathways in combination with an interactive digital technology. For the purposes of this review, interventions were considered “combined” when the neurostimulation and interactive digital technology components were delivered in synchronicity and demonstrated a functional interaction between components. This could be represented in the form of a causal dependency, a closed-loop feedback mechanism, or a shared task context in which both components contributed directly to the same rehabilitative objective. Interventions were not considered “combined” if the modalities were delivered sequentially, in separate sessions, or as independent components of a broader rehabilitation programme without direct interaction. Studies found to be in breach of 1 or more of these criteria were excluded. Therefore, materials containing non-original or non-empirical (e.g., theoretical) data in the form of a review, book, book chapter, protocol, editorial, letter, online registration, or similar were not considered for inclusion. Further, studies were excluded if they were performed in mixed samples where adults with SCI represented less than 50% of the total population or were not accounted for as an independent subgroup. Both for the purposes of comprehensively mapping the research area, and particularly in order to capture the full range of early-stage clinical testing within this emerging field, no restrictions were set upon year of publication, study design, study outcomes, or research methods.

### Screening procedure

Each record was independently double-screened based on the eligibility criteria at 2 consecutive levels. This was performed on the web-based software platform Covidence (24) and was carried out across the author group. To ensure a cohesive screening approach, reviewers performed a pilot screening of the first 25 studies sorted alphabetically by author. Disagreements were discussed and resolved internally before proceeding at the title and abstract screening level. Here, a deliberately overinclusive approach was adopted to ensure that all potentially relevant materials were forwarded for assessment at the full-text screening level. As such, reviewers screened with a broad focus on either interactive digital technologies or stimulation modalities investigated in an SCI context. Mutually forwarded studies were then assessed at the full-text screening level using all eligibility criteria and a narrower focus on a combined intervention. At this stage, corresponding authors were contacted if study information important to the decision-making process required clarification. Reasons for exclusion at the full-text level were documented, and disagreements were again resolved internally via discussion. Studies were tagged and noted if they contained relevant information but were published in an ineligible format. Studies were similarly tagged if they were deemed useful for contextualizing the discussion, for example combined interventions in the general population or in other neurological groups.

### Data extraction

Data from all included studies were independently double-extracted between a combination of the first and second author and a trained assistant into a standardized table covering publication details, sample characteristics, injury details, study setting and design, intervention specifics, outcomes, and relevant findings. Discrepancies in extraction were resolved internally.

### Synthesis methods

**Fig. 1:**
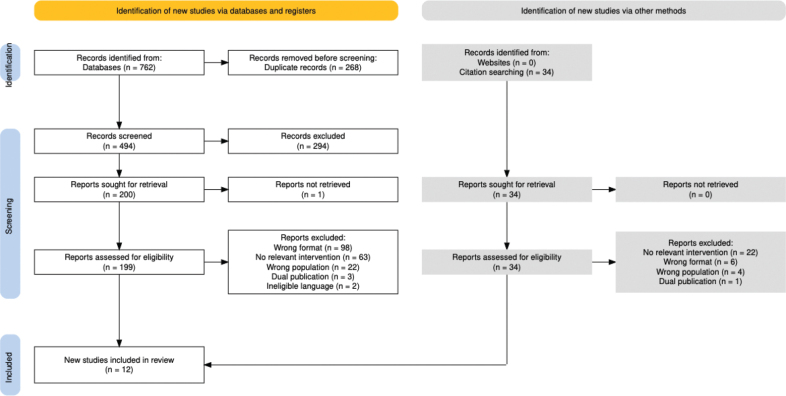
Study flow diagram compliant with the Preferred Reporting Items for Systematic Reviews and Meta-Analyses (PRISMA).

The narrative synthesis was informed by the data extraction tables (Figs S3 and S4), which charted study characteristics such as injury details, intervention specifics, outcomes, and relevant findings. This helped to identify commonalities and other meaningful patterns across the included studies. Equally, we intended for the presentation of results to be intuitive and aligned with the aims and conceptual framework underpinning the review, namely, the combined use of non-invasive neurostimulation modalities and interactive digital technologies in SCI rehabilitation. For this reason, we identified the intervention as the point of comparison. Lastly, we considered the most clinically meaningful way of organizing the results by intervention was to adopt a physiological focus, basing the presentation around the anatomical site of action of the stimulation modality, and the manner in which different interactive digital technologies can modulate or supplement its delivery. An interactive PRISMA-compliant flow diagram (25) was used to visualize the screening process (Fig. 1).

### Critical appraisal of evidence

In accordance with established scoping review methodology, a formal risk-of-bias assessment was not conducted. However, methodological limitations of the included studies were identified through data extraction and narratively presented in a dedicated section of the Discussion.

## RESULTS

### Study flow

The database searches yielded a total of 762 records, of which 268 were identified as duplicates. Therefore, a total of 494 unique records were screened at the title-and-abstract level, where 294 records were excluded. While 200 records were sought for retrieval, 1 study could not be retrieved, and is listed in Fig. S5. The remaining 199 records were screened for eligibility at the full-text level, of which 188 were excluded. Reasons for exclusion comprised records published in the wrong format (*n* = 98, e.g., reviews, editorials, protocols, book chapters, trial registrations), containing no relevant intervention (*n* = 63), conducted in the wrong population (*n* = 22, e.g., non-SCI, mixed cohorts with < 50% SCI participants), representing a dual publication (*n* = 3), and written in an ineligible language (*n* = 2). Resultantly, 11 studies from the database searches were forwarded for analysis. A total of 34 records were identified via the additional searches, of which 33 were removed at the full-text screening level. Reasons for exclusion comprised records containing no relevant intervention (*n* = 22), published in the wrong format (*n* = 6), conducted in the wrong population (*n* = 4), and representing a dual publication (*n* = 1). Therefore, 1 additional record was added to the analysis, meaning a total of 12 records were included in the present review. Fig. 1 visualizes study flow in a PRISMA-compliant diagram (25). Inter-rater agreement was substantial at the title-and-abstract screening stage (Cohen’s κ = 0.61–0.64; agreement = 83.1–83.3%) and moderate to substantial at the full-text screening stage (Cohen’s κ = 0.59–0.60; agreement = 87.5–94.0%). These agreement statistics should be interpreted in light of the deliberately overinclusive screening approach adopted at the title-and-abstract screening level, which was designed to prioritize retrieval sensitivity.

### Study characteristics

Research was most frequently conducted in Canada (*n* = 4) (26–29), the United Kingdom (*n* = 3) (30–32), and Australia (*n* = 2) (33, 34), though the United States (*n* = 1) (35), Spain (*n* = 1) (36), and China (*n* = 1) (37) also featured. Regarding methodological rigour, the evidence base was largely exploratory. Study designs were predominantly small-scale, consisting of 4 case studies or reports (27, 28, 36, 37), 3 pilot studies (31, 32, 35), 2 case series (26, 30), 2 randomized crossover trials (29, 33), and 1 prospective longitudinal study (34). Eight studies lacked independent control groups or conditions (27, 28, 30–32, 34, 36, 37). Individual sample sizes were limited, ranging from 1–13 participants, with a total of 51 unique participants (men = 35 [68.6%]; women = 12 [23.5%]; not reported = 4 [7.8%], aged 21–80 years) included across the 12 studies. This total accounts for 2 publications reporting acute and longitudinal data from the same participant (27, 28). Substantial heterogeneity in injury characteristics was exhibited, with both complete (AIS A) and incomplete (AIS B, C, D) SCIs represented. Equally, neurological injury levels spanned from the upper cervical to the lower thoracic spine, ranging from C1 to T12. While most participants were in the chronic phase of injury (>12 months post-onset), several studies specifically included individuals in the sub-acute phase of injury (ranging from 20 days to 12 months post-injury) (31, 36, 37). Non-invasive neurostimulation techniques comprised NMES/FES (*n* = 11) (26–36) and tSCS (37) (*n* = 1), whereas a broad range of interactive digital technologies were identified across the 12 included studies. These included immersive or non-immersive VR (*n* = 7) (31–37), joystick (27, 28) and balance-controlled videogames (26), and visual feedback exergames integrated within upper-limb workstations (29) and instrumented standing frames (30). Study settings primarily involved clinical environments (26–28, 30–37), though 1 study implemented a home-based tele-rehabilitation model (29).

### Narrative synthesis

Interventions were categorized into 2 separate levels of neurological targeting: peripheral neurostimulation (*n* = 11), which uses NMES/FES to drive muscle-level activation; and spinal-directed neurostimulation (*n* = 1), which uses tSCS to activate neural circuits below the level of injury.

### Category 1: Peripheral neurostimulation

Eleven studies utilized peripheral neurostimulation, specifically NMES/FES, paired with a variety of interactive digital technologies to drive task-specific motor recovery (26–36).

*Gamified task environment with pre-programmed FES* (*n* = 6)*.* In 6 of the included studies, the interactive digital technology provided a gamified, task-oriented training environment with immersive biofeedback to guide functional movements (29–33, 35). Rather than acting as the primary controller, the technology in these studies provided a simulated context for exercise.

In 1 pilot study, 11 participants with SCI presenting with clinically diverse injuries (C1-T12, AIS C-D) received non-immersive VR biofeedback to encourage voluntary effort during FES-supported leg cycling (31). Over 12 sessions of increasing intensity, improvements in motor score (≥ 8 points) were observed for 5 out of 11 participants, with sub-acute participants showing a higher median increase (16%) compared with those in the chronic phase (10%) (31). While 5 participants demonstrated moderate-to-large gains in voluntary power output (up to 14 W; *R*^2^> 0.3–0.7), these gains were positively correlated with their initial voluntary capacity (*R* = 0.50) rather than changes in motor scores (31). Functional independence was improved in 5 out of 11 participants, a trend that was observed almost exclusively in the sub-acute group (31). Subjective feedback recorded in training diaries indicated that participants enjoyed the intervention (31). A subsequent longitudinal pilot study, extended over 12 weeks, found the same custom intervention to be associated with sustained gains in walking speed (−10 s and −3 s on the 10MWT), walking endurance (+33 m and +135 m on the 6MWT), and functional independence (+3 and +1 points on the WISCI-II) for both participants (32). Improvements in balance (incremental BBS scores from 30 to 41 for 1 participant) and trunk control (+4 points on the TIS for 1 participant) were also demonstrated (32). Participants anecdotally reported enjoyment, motivation, and positive distraction during training (32). In a similar experimental set-up with an additional control condition, 8 men with chronic SCI (T4-T12, AIS A-C) achieved significantly higher arm (42%) and leg (23%) activity counts during non-immersive indoor VR-assisted hybrid arm+FES-leg cycling than during outdoor overground hybrid arm+FES-leg cycling (33). Although the VR condition facilitated a greater traversal distance (~1.8x), faster speeds (~2.2x), and significantly higher mechanical efficiency (gross MEG: 4.1%–5.1%; net MEN: 5.4%–6.7%, *p* < 0.05), mean oxygen uptake (VR: 1316 mL/min vs outdoor: 1255 mL/min) and heart rate (VR: 125 ± 3 b/min vs outdoor: 128 ± 3 b/min) remained comparable between conditions (33). Through validated assessments, participants perceived both conditions as exercising “very hard” (RPE 8.1), with no significant differences in psychological engagement, revitalization, or user satisfaction (33). A pilot study comparing the cardiovascular effects of hybrid NMES rowing with and without immersive task-oriented VR demonstrated contrasting results (35). During the VR condition, 2 participants with motor complete paraplegia (T8–T10, AIS B–C) recorded higher exercise heart rate (VR: 122–144 bpm vs No-VR: 108–121 bpm) and percentage of heart rate reserve (VR: 67%–45% vs No-VR: 46.5%–36%) (35). Correspondingly, the VR condition was associated with a higher perceived effort (RPE VR: 9–10 vs No-VR: 7), despite rowing metrics such as distance, stroke rate, and power remaining comparable between conditions, or slightly depressed in the VR condition (35). Additionally, participant showed a much higher increase in systolic blood pressure during the VR condition (18.8 vs 4.5 mm Hg) (35).

Shifting the application of gamified biofeedback away from rhythmic cycling and rowing, a longitudinal 6-month case-series followed 3 men with complete SCI (T2–T4, AIS A) performing FES-assisted standing sessions supported by an instrumented frame (30). The equipment was capable of providing real-time force plate data, delivered via a 2D skiing exergame, to inform participants of their bodyweight distribution and achieve postural goals (30). Results demonstrated high ground reaction forces (70% of bodyweight) on the stimulated leg without fatigue-related decline over the hour-long sessions (30). Across 36–41 total sessions, the average bodyweight supported by the stimulated leg increased significantly from 63.7% to 78.6% (*p* = 0.0071), representing a mean improvement of 13.88 percentage points (30). While the trunk’s range of motion improved from 12.1° to 15.2°, statistical significance was not reached (*p* = 0.0966) (30). Similar task-oriented exercises combining FES with interactive digital technologies have shown potential for restoring fine motor control in the upper extremities. In a 6-week randomized crossover trial, 13 individuals with tetraplegia (C5–C7) delivered FES to their own hand muscles via tooth-click sensors (29). This allowed independent operation of the ReJoyce workstation, outfitted with ergonomic tools designed to mimic objects from daily life, which could be used to control custom 2D videogames (29). Across 30 sessions, the experimental condition was significantly more effective than the control condition (passive FES combined with general strength training) for improving upper extremity functional performance (ARAT) (13.0% vs 4.0%, *p* < 0.01) and arm and dexterous hand function (RAHFT) (16% vs 3.3%, *p* < 0.01), demonstrating large to very large effect sizes in both outcome measures (ARAT *d* = 1.32; RAHFT: *d* = 1.95), which exceeded the thresholds for minimal clinically important difference (29). These improvements were sustained at a 30-week follow-up, and 9 participants (69.2%) indicated a willingness to repeat treatment on their untrained hand (29). Although the grasp and placement tasks produced the most significant improvement in performance, no statistically significant difference was observed for more challenging tasks, such as turning a key or doorknob (29).

*Algorithmic closed-loop control* (*n* = 3)*.* In 3 of the included studies, the interactive digital technology served as an active control loop where biomechanical or neural signals are automatically converted into real-time stimulation commands (26, 34, 36). These interventions made use of autonomous closed-loop systems where sensor data determined how much stimulation was delivered.

In a 52-week longitudinal study involving 4 men with chronic, complete SCI (AIS A–B), a multimodal BCI-FES-VR system, using EEG-based motor imagery to trigger electrical stimulation congruent with participant intent, enabled participants to perform leg cycling on a motorized ergometer while immersed in a simulated natural environment (34). After approximately 111 total training hours, 2 participants sufficiently recovered voluntary muscle contractions below the level of injury to necessitate a reclassification of injury completeness from AIS A/B to AIS C (34). Additionally, all participants demonstrated improved sensory function (touch, vibration, and temperature) and musculoskeletal gains, including reduced muscle fat infiltration and increased bone mineral density (+1.6% at the femoral neck; + 4.2% at the lumbar spine) (34). Furthermore, 1 participant reported recovered autonomic thermoregulation (34). BCI-FES-VR architecture utilizing a similar neural closed-loop framework was also investigated as an approach to restore focal hand function in the case report of a 55-year-old man with sub-acute, incomplete SCI (C5, AIS D) (36). As the participant practised movement attempts, motor-related cortical potentials, detectable via EEG, prompted FES delivery to the forearm (36). As this stimulation was delivered, a VR visualization of a closing hand was presented to the participant (36). After 5 sessions, quantitative prehension (GRASSP) in the intervention arm improved from 20 to 24 points, whereas the control arm, which received conventional occupational therapy, remained stable at 28 points (36). Functional independence (SCIM) increased substantially from 28 to 42 points (36). The participant reported “very, very light” exertion (Borg score 6) during the intervention, which they found to be both engaging and motivating (36).

Moving away from the decoding of neural signals, five individuals with chronic, motor incomplete SCI (C1–T10, AIS C–D) participated in 12 sessions of ankle FES paired with visual feedback balance training (26). Four interactive games, played twice per session, required participants to control their centre-of-pressure (COP) to either maintain a static position or perform weight shifts towards on-screen targets (26). Four participants (80%) demonstrated improvements in balance exceeding 2SD on at least one of three clinical scales, though no clinically meaningful improvements in balance confidence were observed (26). Dynamic stability improved across all participants with available biomechanical data (4/5), with an increase in maximal centre-of-pressure excursion area (7.3%–74.2%) observed immediately post-training and generally maintained at an 8-week follow-up (26). Contrastingly, static postural sway remained largely unchanged, with only two participants demonstrating > 2 SD reductions in mean COP velocity or displacement in specific sway parameters (26). Subjective reports from semi-structured interviews indicated that participants felt both stronger and more independent during the intervention (26).

*User-mediated control within digital interface* (*n* = 2)*.* In 2 of the included studies, the interactive digital technology is supported by a manual interface or controller that allows the user to consciously modulate stimulation parameters in real-time (27, 28).

In 1 case study, a 57-year-old man with chronic, complete SCI (T3–T4, AIS A) navigated a 2D “snake” exergame by performing FES-induced plantarflexion (PF) and dorsiflexion (DF) actions against mechanical resistance (27). The participant used a manual joystick to modulate FES intensity in order to control on-screen action and achieve game-related goals (27). Across approximately 300 successful repetitions of ankle movement during a single session, muscle performance remained stable, with peak torque (PF: 15.3–16.4 Nm; DF: –9.2 to –9.9 Nm) and range of motion (PF: ~19°; DF: –12.5° to –9.4°) showing no significant fatigue-related decline (27). When expanded to a 3 day/week, 48-session programme, the same case participant achieved a number of physiological and performance gains, particularly within the first 25 sessions (28). By the 20th session, the participant was able to sustain FES-induced muscle contractions for the duration of a 45-min trial, having previously lasted 20 min before the onset of fatigue (28). Correspondingly, peak plantarflexion torque increased from ~11 Nm to ~27 Nm, and dorsiflexion torque increased substantially from ~–5 Nm to ~–16 Nm, indicating improved muscle strength (28). Likewise, active range of motion increased from approximately 18° to 29° in plantarflexion and from –3° to –18° in dorsiflexion (28). These physical gains were reflected in significantly improved game performance, with the participant’s score increasing from 12 to 421 points (28). In both studies, open-question interviews revealed that the participant enjoyed the intervention and expressed a willingness to continue treatment (27, 28).

### Category 2: Spinal-directed neurostimulation

One case report utilized spinal-directed neurostimulation, specifically tSCS, supplemented by an immersive VR-based arm and leg cycling interface (37).

A 50-year-old woman sustaining a sub-acute SCI (C4, AIS D) received tSCS to the cervical and lumbosacral regions while pedalling, synchronized to an avatar’s movements, and augmented by real-time performance feedback and virtual competition (37). After 6 weeks (30 min daily, 5 days/week) of training, the participant demonstrated improved ASIA motor scores (52 to 88) and ASIA sensory scores (139 to 169) (37). SCIM-III scores increased from 10 to 82, representing substantial improvements in functional independence, and enabling the participant to walk with aids for over 300 m (37). Physical gains were accompanied by significantly reduced levels of anxiety and depression assessed by standardized testing, and MRI-confirmed amelioration of spinal cord compression (37).

## DISCUSSION

### Summary of findings

This scoping review mapped the available evidence concerning the combined use of non-invasive, neurostimulation techniques and interactive digital technologies in SCI rehabilitation. Twelve unique studies were identified, categorized by the target of the stimulation provided, i.e., peripheral neurostimulation (NMES/FES; *n* = 11) (26–36) and spinal-directed neurostimulation (tSCS; *n* = 1) (37). Despite the broad eligibility criteria and search strategy, no eligible studies combining interactive digital technologies with other non-invasive neurostimulation modalities were identified. While multiple studies evaluated peripheral neurostimulation using a range of protocols and intervention formats, evidence relating to spinal-directed neurostimulation remains highly preliminary, with only a single eligible case report identified. Within these 2 categories, a total of 51 unique participants engaged with interactive digital technologies either as virtual task environments (*n* = 7) (29–33, 35, 37), closed-loop control systems (*n* = 3) (26, 34, 36), or via manual videogame controllers (*n* = 2) (27, 28). Collectively, the findings provided preliminary indications of functional improvements to outcomes such as balance (26, 30, 32), upper-limb dexterity (29, 36), and motor function (31, 32, 37) in response to gross-motor and dynamic tasks, though only limited improvements in fine-motor skills (29) and postural control (26, 30) were evidenced. In 1 instance, neurological recovery was sufficient to warrant AIS reclassification (34). However, the finding comes from a small, non-randomized study with subacute participants, meaning gains cannot be definitively attributed to the intervention alone. Preliminary findings also suggest that the level of digital immersion can modulate physiological strain (33, 35). While immersive VR rowing was associated with higher cardiovascular output and heart rate reserve (35), non-immersive VR cycling elicited heart rate demands comparable to conventional exercise (33). However, this may also be impacted by the stable indoor environment supporting continuous, high-volume output, as opposed to the cautious manoeuvring often required when traversing outdoor terrain. Increases in biomechanical output, such as torque production (27, 28, 30), voluntary power output (31, 32), and muscle composition (34) were observed during combined interventions. However, fatigue-related decline was observed in single cases (26, 28). Nevertheless, participants frequently perceived combined interventions as motivating and enjoyable (26–28, 31, 32, 35, 36) and expressed an interest to continue often high-intensity treatment beyond study termination (26–29). As such, the combination of these 2 approaches may be beneficial for individuals with SCI as a rehabilitation strategy and as a means of enhancing patient engagement and adherence. However, findings in sub-acute groups should be interpreted cautiously alongside spontaneous recovery (31, 36, 37). Equally, the field is still developing and is characterized by small-scale studies with limited external validity, placing low in the evidence hierarchy.

### Study findings in context

Multiple SCI studies met several of the eligibility criteria but did not fulfil all requirements for inclusion, thereby representing unique points of comparison. For example, several studies have combined non-invasive neurostimulation techniques with passive, digital content, including pre-recorded 3D videos (38–41) and illusion-based paradigms (42–45). In 1 example, transcranial direct current stimulation (tDCS) combined with pre-recorded VR was associated with reductions in neuropathic pain intensity and interference (42). In 2 further examples, investigators designed their VR experience to successfully complete motor imagery commands irrespective of performance in order to positively reinforce participants (40, 41).

Design studies reporting pre-clinical data used to evaluate or validate system architecture, performance, or feasibility were also excluded from the review (46–49) to maintain conceptual clarity. Nevertheless, these studies reflect technological innovations within the field and represent an important translational pipeline through which engineering precedes clinical implementation. As such, monitoring the progression of these systems into clinical SCI trials will be important for understanding how the field matures. On the topic of technological advancement, proprioceptive and tactile stimulation modalities, such as haptic gloves and sleeves (50, 51) known for targeting sensorimotor feedback mechanisms rather than neuromodulation, were also excluded from our study. Through afferent sensory enrichment, these devices have been shown within the SCI literature to improve embodiment while situated in VR environments (41, 51, 52). This is subtly different from the central aim of our included stimulation techniques, which through efferent neuromodulation are primarily designed to induce muscle contractions and support functional performance. Nevertheless, the existence of interventions combining haptic feedback with interactive digital technologies is further proof of the movement towards multisensory, technology-mediated rehabilitation strategies within the SCI literature.

Further, studies evaluating an interactive digital technology and a non-invasive stimulation modality as independent or sequential components of a broader rehabilitation programme were excluded from the present review (53–56). In such examples, improvements in AIS classification, lower limb motor scores, trunk control, and ambulatory function are observed, though the evidence is largely derived from case reports (53–55), thereby limiting external validity. Similarly, at least 1 randomized trial evaluated VR and FES as comparator adjuncts to conventional physical therapy, finding that both components improved sitting balance for individuals with SCI (57). Although their synergistic effects cannot be determined from such research inquiries, both immersive digital technologies and non-invasive stimulation appear capable of supporting functional gains as independent interventions within SCI rehabilitation.

Lastly, studies combining implanted stimulation devices with interactive digital technologies to achieve task-specific training in SCI rehabilitation were excluded (58, 59). The 2 interconnected examples identified by our search were associated with improvements in mobility, walking ability, strength, bladder function, and spasticity (58, 59). As such, though outside our scope, the combination of interactive digital technologies with percutaneous stimulation remains viable.

### Limitations of the included studies

Several limitations of the included studies were identified. First, the current evidence base must be interpreted as preliminary and exploratory. The collective findings were drawn heavily from case studies and reports (27, 28, 36, 37), case series (26, 30), and small-scale pilot studies (31, 32, 35), all with limited generalizability. Moreover, heterogeneity across the 12 included studies complicates the interpretation of findings. Task-specific effects were pronounced, as demonstrated by improvements being largely restricted to the gross motor or dynamic tasks practised, rather than those targeting static postural control and fine-motor skills (26, 29). Outcomes were also driven by dose–response relationships. For instance, while 4-week protocols resulted in initial motor gains (26, 31), only the 52-week intervention resulted in AIS reclassification (34). Without appropriate control conditions, it remains unclear to what extent these changes can be causally attributed to the intervention rather than to natural recovery processes. Given that most AIS conversion and motor recovery occurs within the first 6–9 months post-injury, and that motor recovery is most pronounced within the first 3 months (60), future randomized controlled trials are needed to establish a baseline rate of natural improvement for comparison with sub-acute experimental groups. Control groups may also help to determine the efficacy of combining interactive digital technologies with non-invasive neurophysiological stimulation as opposed to conventional or individual treatment. Furthermore, intervention modality influenced response intensity, with immersive VR conditions facilitating higher cardiovascular demands and perceived exertion compared with non-VR conditions (33, 35). As an additional methodological critique, 1 included crossover study conceded that baseline scores were elevated after the first treatment, indicating carryover effects before the start of the second treatment (29). Future crossover studies in the area should carefully consider the length of their washout period between treatments. Notably, all included studies regarded functional and biomechanical improvements as proxies for neurological recovery. While functional gains are arguably the most critical outcomes from a clinical and patient-centred perspective, in the absence of brain/spinal cord imaging or measurement of motor evoked potentials, there is a lack of direct physiological confirmation of neuroplasticity resulting from the combined interventions. In a similar vein, evidence to support the psychological benefits from the combined interventions was mostly anecdotal, with only a few studies using validated scales to assess psychological states (26, 33, 37).

### Limitations of this review

There are inherent limitations associated with our review framework. In choosing to perform a narrative synthesis, the findings reported here necessarily rely on the results as presented in the included studies. However, in fields such as SCI rehabilitation, even large, well-conducted trials often struggle to demonstrate clear treatment effects (61). Because scoping reviews aim to map the existing literature, a formal risk-of-bias assessment was not performed to critically appraise the quality of evidence. As such, this approach may give the impression of a stronger evidence base than can currently be supported by demonstrably high-quality clinical trials. Another limitation of this review is the potential exclusion of relevant studies due to inconsistent reporting of equipment capabilities. To maintain a transparent and reproducible process, we decided that no technology would be assumed to be “interactive” unless described as such in the source material. For example, operation of the ReJoyce workstation in Kowalczewski et al. (29) involved an interactive, digital component that was lacking in other reporting of the system (62, 63). The principle of remaining faithful to the information reported was applied in cases where insufficient detail was provided to ascertain whether digital technologies functioned in an interactive manner (64, 65). In these cases, records were also excluded. Further, relevant studies published in languages other than English, Danish, Norwegian, or Swedish may similarly exist, but were beyond the scope of the present review. As such, there may exist additional studies that were not included in the present work.

### Clinical implications and avenues for future research

Future research should address the observed methodological limitations of the included studies. For instance, a baseline rate of spontaneous recovery can be established in sub-acute SCI trials with the creation of a control group, which would allow researchers to more precisely attribute functional and neurological recovery to the intervention itself. However, recruitment of sufficiently large SCI samples can be challenging due to the relatively small patient population and the clinical and logistical burden placed on participants (66). Crossover trials should consider washout periods of >1 month to avoid carryover effects, and more large-scale, high-quality trials with long-term follow-up periods are required to determine longitudinal viability and to improve the evidentiary standard of a research area dominated by case reports, pilot studies, and other exploratory insights.

In conclusion, preliminary evidence of combined non-invasive neurostimulation and interactive digital technologies was associated with improved functional outcomes, such as dynamic balance, upper-limb dexterity, and functional independence, and, in 1 case, clinical markers putatively associated with neurological recovery, such as injury completeness. In sub-acute cases, this combination has also been associated with reduced levels of anxiety and depression. Based on the low evidentiary standard of the included studies, these findings are insufficient to support clinical recommendations. Nevertheless, as reported in several studies, participants enjoyed and were highly motivated by the combination, often expressing a willingness to continue treatment. As such, gamification, biofeedback, and immersive settings may support adherence during often high-volume, repetitive exercises. Despite this, clinicians are still recommended to monitor for muscular fatigue. Likewise, as combined interventions were more limited for rehabilitation of fine-motor skills and static postural control, application should be task-specific and tailored to the individual’s needs. Additionally, there are several challenges to implementing complex and resource-intensive interventions into clinical practice. For example, in 1 included study, a 60-min standing session required its own 60-min window for set-up and termination (30), which may not be feasible in busy clinical settings. Likewise, the infrastructure of several studies included highly advanced, custom-built, or novel hardware (30, 34), the complexity of which goes beyond the standard training of most clinicians. As such, a cost–benefits analysis may be required before investing in such technology. However, the included studies also propose avenues for translation into routine practice. For instance, tele-rehabilitation protocols enabled by remote monitoring and affordable workstations demonstrated the feasibility of delivering home-based therapy (29), thereby reducing the travel burdens placed upon participants (26). Furthermore, the use of dry-electrode EEG headsets and custom garments have been demonstrated to reduce setup times to under 15 min, making the intervention more convenient in clinical practice (34). Lastly, the increasing popularity of commercially available VR headsets and stationary trainers in rehabilitation contexts is designed to promote accessibility, as is the move from expensive, highly specialized, and custom robotic systems into consumer-grade applications (31, 33, 35).

## Supplementary Material


