# Supplementary material for: THE COMBINED USE OF NON-INVASIVE NEUROSTIMULATION WITH INTERACTIVE DIGITAL TECHNOLOGIES IN SPINAL CORD INJURY REHABILITATION: A SCOPING REVIEW
Source: J Rehabil Med. 2026 Jul 21;58:45747. doi: 10.2340/jrm.v58.45747 (PMC13397339; doi:10.2340/jrm.v58.45747)
Supplement: Supplementary file 1 [file JRM-58-45747-s1.pdf]

Supplementary Material

Fig. S1. Preferred Reporting Items for Systematic reviews and Meta-Analyses extension for Scoping Reviews (PRISMA-ScR) Checklist

Preferred Reporting Items for Systematic reviews and Meta-Analyses extension for Scoping Reviews (PRISMA-ScR) Checklist

SECTION	ITEM	PRISMA-ScR CHECKLIST ITEM	REPORTED ON PAGE #
**TITLE**			
Title	1	Identify the report as a scoping review.	
**ABSTRACT**			
Structured summary	2	Provide a structured summary that includes (as applicable): background, objectives, eligibility criteria, sources of evidence, charting methods, results, and conclusions that relate to the review questions and objectives.	
**INTRODUCTION**			
Rationale	3	Describe the rationale for the review in the context of what is already known. Explain why the review questions/objectives lend themselves to a scoping review approach.	
Objectives	4	Provide an explicit statement of the questions and objectives being addressed with reference to their key elements (e.g., population or participants, concepts, and context) or other relevant key elements used to conceptualize the review questions and/or objectives.	
**METHODS**			
Protocol and registration	5	Indicate whether a review protocol exists; state if and where it can be accessed (e.g., a Web address); and if available, provide registration information, including the registration number.	
Eligibility criteria	6	Specify characteristics of the sources of evidence used as eligibility criteria (e.g., years considered, language, and publication status), and provide a rationale.	
Information sources*	7	Describe all information sources in the search (e.g., databases with dates of coverage and contact with authors to identify additional sources), as well as the date the most recent search was executed.	
Search	8	Present the full electronic search strategy for at least 1 database, including any limits used, such that it could be repeated.	
Selection of sources of evidence†	9	State the process for selecting sources of evidence (i.e., screening and eligibility) included in the scoping review.	
Data charting process‡	10	Describe the methods of charting data from the included sources of evidence (e.g., calibrated forms or forms that have been tested by the team before their use, and whether data charting was done independently or in duplicate) and any processes for obtaining and confirming data from investigators.	
Data items	11	List and define all variables for which data were sought and any assumptions and simplifications made.	
Critical appraisal of individual sources of evidence§	12	If done, provide a rationale for conducting a critical appraisal of included sources of evidence; describe the methods used and how this information was used in any data synthesis (if appropriate).	
Synthesis of results	13	Describe the methods of handling and summarizing the data that were charted.	

SECTION	ITEM	PRISMA-ScR CHECKLIST ITEM	REPORTED ON PAGE #
**RESULTS**			
Selection of sources of evidence	14	Give numbers of sources of evidence screened, assessed for eligibility, and included in the review, with reasons for exclusions at each stage, ideally using a flow diagram.	
Characteristics of sources of evidence	15	For each source of evidence, present characteristics for which data were charted and provide the citations.	
Critical appraisal within sources of evidence	16	If done, present data on critical appraisal of included sources of evidence (see item 12).	
Results of individual sources of evidence	17	For each included source of evidence, present the relevant data that were charted that relate to the review questions and objectives.	
Synthesis of results	18	Summarize and/or present the charting results as they relate to the review questions and objectives.	
**DISCUSSION**			
Summary of evidence	19	Summarize the main results (including an overview of concepts, themes, and types of evidence available), link to the review questions and objectives, and consider the relevance to key groups.	
Limitations	20	Discuss the limitations of the scoping review process.	
Conclusions	21	Provide a general interpretation of the results with respect to the review questions and objectives, as well as potential implications and/or next steps.	
**FUNDING**			
Funding	22	Describe sources of funding for the included sources of evidence, as well as sources of funding for the scoping review. Describe the role of the funders of the scoping review.	

JBI = Joanna Briggs Institute; PRISMA-ScR = Preferred Reporting Items for Systematic reviews and Meta-Analyses extension for Scoping Reviews.
* Where *sources of evidence* (see second footnote) are compiled from, such as bibliographic databases, social media platforms, and Web sites.
† A more inclusive/heterogeneous term used to account for the different types of evidence or data sources (e.g., quantitative and/or qualitative research, expert opinion, and policy documents) that may be eligible in a scoping review as opposed to only studies. This is not to be confused with *information sources* (see first footnote).
‡ The frameworks by Arksey and O'Malley (6) and Levac and colleagues (7) and the JBI guidance (4, 5) refer to the process of data extraction in a scoping review as data charting.
§ The process of systematically examining research evidence to assess its validity, results, and relevance before using it to inform a decision. This term is used for items 12 and 19 instead of "risk of bias" (which is more applicable to systematic reviews of interventions) to include and acknowledge the various sources of evidence that may be used in a scoping review (e.g., quantitative and/or qualitative research, expert opinion, and policy document).

From: Tricco AC, Lillie E, Zarin W, O'Brien KK, Colquhoun H, Levac D, et al. PRISMA Extension for Scoping Reviews (PRISMAScR): Checklist and Explanation. Ann Intern Med. 2018;169:467–473. doi: 10.7326/M18-0850.

Fig. S2. Final search string, entered into seven databases on 6th January 2026

**Population Block**	**Intervention 1 Block**	**Intervention 2 Block**
Spinal cord injury (SH) wo/e	Virtual reality (SH) wo/e	Stimulation (SH) wo/e
Spinal cord lesion (SH) wo/e	Augmented reality (SH) wo/e	"Stimulat*"
Quadriplegia (SH) wo/e	Gamification (SH) wo/e	"FES"
Paraplegia (SH) wo/e	"Virtual realit*"	"NMES"
Paralysis (SH) wo/e	"Augmented realit*"	"TENS"
Spinal cord injuries (SH) wo/e	"Extended realit*"	"TES"
Spinal injuries (SH) wo/e	"Mixed realit*"	"EMS"
Spine injury (SH) wo/e	"Game*"	"tSCS"
"Spinal cord injur*"	"Gami*"	"TMS"
"Spinal cord lesion*"	"Videogam*"	"rTMS"
"Spinal cord trauma*"	"Exergam*"	"PMS"
"Spinal cord infarction*"	"Immersive environment*"	"tDCS"
"Spinal injur*"	"Virtual environment*"	"tACS"
"Spinal lesion*"		"tRNS"
"Spinal trauma*"		
"Spine injur*"		
"Tetraplegi*"		
"Paraplegi*"		
"Quadriplegi*"		
"Paraly*"		

Key:
SH = Subject heading
wo/e = Without explosion

* = Truncation operator

Fig. S3. Extraction of study characteristics

Author(s)	Country of Origin	Sample Size	Sample Characteristics	Injury Characteristics	Study Setting	Study Design	Equipment	Study Outcome(s)	Other
Bayon-Calatayud et al. (2017)	Spain	N = 1	Age: 55<br>Sex: Male (100%)	Level: C5<br>Completeness: Incomplete (AIS D)<br>Time since injury: 4 months	Clinical	Case study. Mixed (quantitative and qualitative)	*IDT*: VR<br>*Stim*: FES (forearm muscles)	SCIM scale (ADL performance), GRASSP (hand grasping), Borg scale (exertion), usability questionnaire, anecdotal evidence	
Chu et al. (2024)	China	N=1	Age: 50<br>Sex: Female (100%)	Level: C4.<br>Completeness: Incomplete (AIS D).<br>Time since injury: sub-acute (approx.. 20d post-injury at department transfer).	Clinical	Case report. Quantitative	*IDT*: VR<br>*Stim*: tSCS (cervical and lumbosacral regions).	ASIA Motor/Sensory scores, Hamilton Anxiety/Depression (HAMA/HAMD), SCIM-III.	
Duffell et al. (2019)	UK	N=11	Age: Mean (range) = 56.5 (21-80).<br>Sex: Male = 10 (90.9%); Female = 1 (9%).	Level: C1-T12.<br>Completeness: Incomplete (AIS C = 8, or D = 3).<br>Time since injury:<br>5 sub-acute (<12 months); 6 chronic (>12 months)	Clinical	Single-group pilot study. Quantitative	*IDT*: VR<br>*Stim*: FES (quadriceps, hamstring, gluteal muscles)	ISNC-SCI motor score, Oxford scale motor power grading, MAS (lower limb spasticity), SCIM (independence), WISCI II (walking index), 10m-walk test, power output	
Hasnan et al. (2024)	Australia	N=8	Age: Mean = 49.9 ± 5.5.<br>Sex: Male = 8 (100%)	Level: T4–T12.<br>Completeness: Chronic (AIS A, B, and C), with intact lumbosacral lower motor neuron and good hand function.<br>Time since injury: 9.1 years ± 1.5 years.	Clinical	Randomized crossover trial. Quantitative	*IDT*: VR<br>*Stim*: FES (quadriceps, hamstring, gluteal muscles)	Oxygen uptake (VO2), energy expenditure, heart rate, rate of perceived exertion, activity counts (Actigraph), user satisfaction and self-reports (QUEST	

								2.0, EIFI), VR symptoms (VRSQ), activation or arousal state after the cooling down period post-exercise (AD-ACL)	
Heidorn et al. (2026)	USA	N=2	Age: P1 = 23; *P2 = 57*<br>Sex: Male = 2 (100%)	Level: P1 = T8; *P2 = T10*<br>Completeness: P1 = AIS B; *P2 = AIS C.*<br>Time since injury: P1 = 4 years; *P2 = 5 years*	Clinical	Pilot, proof-of-concept study.<br>Mixed (quantitative and qualitative)	*IDT:* VR<br>*Stim:* NMES (Quadriceps, hamstrings, gluteal and sartorius muscles)<br><br>*P1 used an implanted 12-channel stimulator-telemeter; *P2 used a custom 8-channel surface stimulator - P2 is the only participant who utilized non-invasive NMES exclusively)*	Heart rate (resting, average exercising, peak), heart rate reserve (HRR%), systolic/diastolic blood pressure, rowing stroke rate, distance (m), rowing power (W), rating of engagement (1–5 scale), Rating of Perceived Exertion (RPE; 1–10 scale)	
Houston et al. (2020)	Canada	N=5	Age: Range = 55–69<br>Sex: Male = 1 (20%), Female = 4 (80%)	Level: C1, C3, C5, T6, T10.<br>Completeness: AIS C (N=3), AIS D (N=2).<br>Time since injury: Chronic (22–97 months)	Clinical	Case series (single-subject experimental design).<br>Mixed (quantitative and qualitative)	*IDT:* Visual feedback balance training via computer monitor<br>*Stim:* FES (plantarflexors, dorsiflexors)	Berg Balance Scale (BBS), mini-Balance Evaluation Systems Test (mini-BESTest), Activities-specific Balance Confidence (ABC) Scale, postural sway, limits of stability test during standing, user acceptability	
Kowalczewski et al. (2011)	Canada	N=13	Age: Mean = 35.9 ± 12.0<br>Sex: Male = 7 (53.8%)<br>Female = 6 (46.2%)	Level: Ranged from C5, C6, C7<br>Completeness: 31% complete (N=4)<br>Time since injury: Mean = 3.6 ± 2.1	Mixed (home and lab-based)	Randomized crossover trial.<br>Mixed (quantitative	*IDT*: Videogames displayed on a laptop screen connected to the ReJoyce workstation<br>*Stim*: FES (hand muscles)	Hand function (Action Research Arm Test), arm and hand function (ReJoyce Arm Hand Function Test), grasp	

						and qualitative)		and pinch force (standard physiotherapy dynamometers).	
Massey et al. (2025)	UK	N=2	Age: Not reported<br>Sex: Not reported	Level: C1, T9<br>Completeness: AIS D (N=2)<br>Time since injury: Chronic (>1 year)	Clinical	Pilot study. Mixed (quantitative and qualitative)	*IDT*: VR<br>*Stim*: FES	International Standards for Neurological Classification of SCI (ISNCSCI), 10- meter walk tests (10MWT), 6- minute walk tests (6MWT), trunk impairment scale (TIS), Berg Balance scale (BBS), Walking Index for SCI (WISCI- II), voluntary cycling power, user experience.	
Pizzolato et al. (2024)	Australia	N=4	Age: Mean (range) = 40.5 (36-48).<br>Sex: Male: 4 (100%).	Level: C4, T1, C4, C6.<br>Completeness: AIS A (N=3), AIS B (N=1).<br>Time since injury: Range = 3-15 years, Mean = 8.8 years	Clinical	Prospective, longitudinal, single-arm intervention al study. Quantitative	*IDT:* VR<br>*Stim*: FES (quadriceps, hamstrings, calf muscles)	AIS grade, quantitative sensory testing, bone mineral density, muscle quality (MRI), self-reported changes in thermoregulation.	
Sayenko et al. (2011)	Canada	N=1	Age: 57<br>Sex: Male = 1 (100%)	Level: T3-T4.<br>Completeness: Complete (AIS A).<br>Time since injury: 4 years.	Clinical	Case study Mixed (quantitative and qualitative)	*IDT*: Videogame controlled via joystick and ankle motions.<br>*Stim*: 4-channel NMES delivering transcutaneous FES (plantarflexors and dorsiflexors).	NMES intensity, duration of stimulation intervals for each muscle group, overall torque representing resultant torque exerted by the stimulated	

								muscles, passive torque produced by the training device, angular displacement of the foot platform, open-question interview (capturing motivational aspects, proposed improvements to training system)	
Sayenko et al. (2009)	Canada	N=1	Age: 57<br>Sex: Male = 1 (100%)	Level: T3-T4.<br>Completeness: Complete (AIS A).<br>Time since injury: 4 years.	Clinical	Case study Mixed (quantitative and qualitative)	*IDT*: Videogame controlled via joystick and ankle motions.<br>*Stim*: 4-channel NMES delivering transcutaneous FES (plantarflexors and dorsiflexors).	Intensity and duration of FES<br>for each muscle group, angular displacement of the foot, torque produced in the ankle joints, open-question interview (capturing motivational aspects, proposed improvements to training system)	
Zoulias et al. (2019)	UK	N=3	Age: Mean (range) = 34.3 (33-36)<br>Sex: Male = 3 (100%).	Level: T2, T4, T4<br>Completeness: Complete (AIS A).<br>Time since injury: 1, 4, 15 years. Mean = 6.7 years	Clinical	Case series. Quantitative	*IDT*: Instrumented frame with "exergame"<br>*Stim*: FES (gluteul muscles, calves, and quadriceps)	Ground reaction forces (GRF), body weight distribution, range of motion (Clavicle marker), standing time	

Fig. S4. Extraction of study interventions and main results

Author(s)	Study Intervention	Main Result(s)
Bayon-Calatayud et al. (2017)	• BCI-FES-VR training: 5 sessions (~60 min each) over a 10-day period.<br>• Structure: Blocks of 20 trials; participant seated facing a virtual hand display.<br>• Sequence: 10s rest period followed by 3s Movement Attempt (MA) phase.<br>• Synchronous Trigger: BCI detection of MA intention triggered 2s of FES to forearm muscles and corresponding VR feedback for hand grasping.<br>• Concurrent Therapy: 1h of standard bilateral occupational therapy; BCI-FES-VR applied only to the intervention arm.	• Quantitative (Function): SCIM score (ADLs) improved from 28 to 42; GRASSP prehension score in the intervention arm increased from 20 to 24 (no change in control arm).<br>• Quantitative (System): Mean BCI classification accuracy was 85.8 ± 11.8%.<br>• Qualitative (Experience): Scored as "very, very light" exertion (Borg = 6); rated as more engaging and motivating than previous exercises
Chu et al. (2024)	• Combined VR-Cycling + tSCS: 30 min/session, 5 days/week for 6 weeks.<br>• IDT (VR Cycling): Active arm and leg cycling (DN-813 device) synchronized with VR glasses (Lei Niao Air Plus).<br>• VR Feedback: Real-time metrics (distance, duration, calories, laps) and competitive environment with virtual co-riders.<br>• VR Customization: Therapist-controlled weather (sunny, cloudy, snowy) and time of day (sunrise, noon, sunset).<br>• Stimulation parameters: 30 Hz frequency, 0.2 ms biphasic rectangular pulses, intensity 60–80 mA.<br>• Concurrent Therapy: Conducted daily following routine rehabilitation (respiratory training, NMES, limb integration).	• Neurological (ASIA) - Motor scores: Increased from 52 to 88; Sensory scores: Increased from 139 to 169; Improvement was significantly more pronounced in the lower extremities than the upper limbs.<br>• Functional (ADLs) - SCIM-III: Increased from 10 to 82 points; Mobility: Progressed from minimal bed mobility to walking 300 meters independently with a walking aid.<br>• Muscle Strength - Overall: Limb strength reached levels 3–5 on the manual muscle testing scale; Hand: Specific improvements in the middle finger flexor and little finger abduction, which improved from grades 0–2 (minimal/absent) to grade 3.<br>• Psychological - Significant reductions in both anxiety (HAMA) and depression (HAMD) scores were observed as physical function improved.<br>• Imaging (MRI) - Follow-up MRI showed an amelioration (reduction) in spinal cord compression.<br><br>SEE FIGURE 4
Duffell et al. (2019)	• iCycle Training (FES + VR Biofeedback): 3 sessions/week for 4 weeks (12 total).<br>• Structure: Session duration increased from 20 to 45 min. Includes warm-up (passive → FES), up to 5 velodrome "races" (with 2–3 min rest), free cycling, and cool-down.<br>• Interface (Biofeedback): Speed of the virtual avatar is driven by voluntary torque, measured on alternate revolutions (non-stimulated) to separate voluntary effort from FES.<br>• Stimulation parameters: 30 Hz frequency, 200 μs pulse width; intensity adjusted to tolerance (max muscle contraction without overflow).	• Neurological (Motor Function) - ISNC-SCI Motor Score: Median improvement was 8.0 points for sub-acute and 3.5 points for chronic participants. Clinical significance (improvement ≥8 points) was reached by 5/11 participants (4 sub-acute, 1 chronic).<br>• Biomechanical (Cycling Power) - Voluntary Power Output (PO): 5/11 participants showed moderate increases (up to 14W). Improvements correlated with baseline cycling ability (R2 = 0.50) but were unrelated to changes in motor scores.<br>• Functional (ADLs & Walking) - SCIM: Improved in 5/11 participants (4 sub-acute, 1 chronic). Walking: Meaningful gains in one sub-acute participant (#15): WISCI-II increased by 5 points and 10m-walk time improved from 82s to 41s.

	• Motivation: Real-time feedback, terrain-based resistance (inclines), and racing against "ghost" avatars of previous performances.	• Muscle Strength & Spasticity - Oxford Scale: Small median increases in knee extension (1.0) and flexion/ankle (0.5). MAS (Spasticity): Minimal median reductions (-0.5) in quadriceps and calves; responses were inconsistent across the sample.<br>• Qualitative (Anecdotal Reports) - Participants reported improved sleep (n=4), improved sensation (n=4), and better standing stability in a standing frame (n=5). The VR competition was noted as highly motivating.<br>• Predictors of Recovery - Improvements were not significantly correlated with age, time since injury, baseline motor scores, training duration, or stimulation intensity.<br><br>SEE FIGURE 3 AND TABLES 2, 3, 4, AND 5
Hasnan et al. (2024)	• Hybrid Cycling (Outdoor vs. Indoor VR): Acute crossover comparison of two conditions.<br>• Structure: 30 min continuous cycling at self-selected "best effort".<br>• IDT (Indoor VR): Berkelbike mounted on Tacx i-Magic stationary trainer. Displays a non-immersive VR simulation (1080p video + GPS data) of the actual outdoor track on a 150cm screen.<br>• Control (Outdoor): Same Berkelbike used on a 1.26 km paved track with 19.4m elevation change.<br>• Stimulation parameters: 35 Hz frequency; intensity (amplitude) independently adjusted by participant up to 150 mA.<br>• User Control: Participants self-selected speed, gearing, and stimulation intensity in both conditions.	• Physiological (Cardiorespiratory) - Oxygen Uptake (VO2): No significant differences in mean (Indoor: 1316 mL/min; Outdoor: 1255 mL/min) or peak VO2. Heart Rate (HR): No significant difference in average (Indoor: 125 bpm; Outdoor: 128 bpm) or peak HR.<br>• Performance (Cycling Metrics) - Distance: 1.8x greater indoors (6980m vs 3826m). Speed: 2.2x faster indoors (231.3 vs 105.5 m/min). Power Output: Significantly higher indoors (21.6W vs 9.3W), though noted as a proxy estimate for the outdoor condition.<br>• Biomechanical (Efficiency & Activity) - Limb Activity: Significantly higher counts indoors for both arms (+42%) and legs (+23%). Mechanical Efficiency: Significantly greater indoors for both Gross (MEG) and Net (MEN) efficiency. Stress/Strain: Net O2 cost and Physical Cost Index were significantly higher outdoors, reflecting lower efficiency.<br>• Subjective (Psychological & Safety) - Perceived Exertion (RPE): No differences; both conditions reached ~8.1/10 ("very hard") by 30 minutes. Mood/Engagement: No significant differences in EIFI (engagement, tranquility) or AD-ACL (energy, tension). Satisfaction & Safety: High satisfaction (QUEST 2.0) with no reported nausea or cybersickness (VRSQ) in the VR condition.
Heidorn et al. (2026)	• Hybrid NMES Rowing (VR vs. No-VR): Acute comparison of rowing with and without immersive VR.<br>• Structure: 4 bouts of 5-min exercise with 10-min rest intervals.<br>• Participants rowed to keep pace with a virtual goose; utilized head-tilting for lane changes and "speed boosts" for strokes >80% of max length.<br>• Interface: A string potentiometer on the ergometer handle triggered stimulation phases (catch, drive, finish, recovery) in real-time based on handle position.	• Physiological (Cardiovascular) - Heart Rate (HR): Consistent tendency for higher average HR during VR for both P1 (+14 bpm) and P2 (+23 bpm). Heart Rate Reserve (HRR): VR increased HRR% for both participants (P1: 36% to 45%; P2: 46.5% to 67%). Blood Pressure: Systolic BP increases were more pronounced following VR sessions, particularly for P1 (+18.8 vs +4.5 mm Hg).<br>• Exercise Intensity - For P1, VR shifted intensity from "light" to "moderate." For P2, VR facilitated a shift from "moderate" to "vigorous" intensity (HRR >60%).<br>• Performance (Rowing Metrics) - Distance: Remained stable for P2 (~449m) but decreased by ~30m for P1 during the VR condition. Stroke Rate: Remained generally stable across conditions, ranging from ~17 to 20 strokes/min. Power Output: P1 showed a slight decrease in power during VR (32W vs 36.5W), while P2 remained stable (~20W).<br>• Subjective (Psychological) - Perceived Exertion (RPE): Reported as higher in the VR condition for both participants (P1: 9/10 vs 7/10; P2: 10/10 vs 7/10). Engagement: Rated very high (4/5 to 5/5) for both conditions; P1 reported a slight preference for VR (5/5) over No-VR (4/5).

Houston et al. (2020)	• FES + Visual Feedback Balance Training (VFBT): 12 sessions (3/week for 4 weeks).<br>• Structure: 1h total: 15m thresholding, 5m harness setup, 5m calibration, 20m active VFBT, and 15m rest.<br>• Interface (VFBT): Participants stood in a harness receiving real-time COP feedback (red dot) on a screen while performing 4 tasks (Bullseye, Hunting, Ellipse, Color Matching).<br>• Stimulation parameters: 40 Hz frequency; 300 µs pulse duration; intensity fluctuated between minimal contraction and 80% of maximal tolerable threshold.<br>• Interaction: A closed-loop proportional-and-derivative (PD) controller adjusted stimulation in real-time based on COP position/velocity to assist target reaching.	• Clinical Balance - BBS: Improvements >2 SD in 3/5 participants; clinically meaningful gains (MDC 4.4) in 4/5 participants at follow-up. mini-BESTest: Improvements >2 SD in 3/5 participants; clinically meaningful gains (MDC 4.67) in 2/5 participants.<br>• Biomechanical (Dynamic Stability) - Limits of Stability (LOS): All 5 participants (100%) showed increased maximal COP excursion area post-training, ranging from 7.3% to 74.2% above baseline. Gains were maintained or further increased at 8-week follow-up (up to 88–90.9% for P4 and P5).<br>• Biomechanical (Postural Sway) - Static Balance: Minimal effect on quiet stance. Only one participant (P1) showed a >2 SD reduction in AP COP velocity; only one participant (P3) showed a >2 SD reduction in ML RMS displacement.<br>• Psychological (Confidence) - ABC Scale: Improvements >2 SD in 2/5 participants. However, none of the participants reached the threshold for clinically meaningful change (MDC 14.87%).<br>• Qualitative (Acceptability) - Participants highly valued the "safe environment" provided by the harness and research team to practice challenging tasks. FES was described as "reawakening" muscles. All participants desired a longer intervention (suggesting 18–24 sessions total).<br>• Data Integrity & Limitations - Biomechanical data were missing for Participant 2 due to fatigue. Isolated technical errors resulted in missing backward COP excursion data for P3 and P5 at baseline and/or follow-up.
Kowalczewski et al. (2011)	• In-Home Tele-therapy (IHT): Crossover RCT; 1 h/d, 5 d/wk for 6 weeks per treatment.<br>• IDT-Stim (ReJoyce ET): Task-oriented workstation (ReJoyce) with 6 custom games (e.g., driving, pouring, boxing).<br>• Interface/Trigger: Wireless tooth-click sensor (earpiece accelerometer) allowed the user to cycle through stimulation states (opening → grasp → off).<br>• Control (Conventional ET): Three 20-min segments: 1) Strength (wrist weights/Powerweb); 2) Accuracy (computer games with a Trackball mouse); 3) TES (passive, cyclical stimulation every 5s).	• Clinical Function (ARAT) - ReJoyce ET: Improved by 13.0% ± 9.8%. Conventional ET: Improved by 4.0% ± 9.6%. Relevance: Only ReJoyce ET exceeded the 10% MCID (Minimal Clinically Important Difference).<br>• Automated Function (RAHFT) - ReJoyce ET: Improved by 16.9% ± 8.6%. Conventional ET: Improved by 3.3% ± 10.2%. Sub-tasks: Greatest gains were seen in grasping and placement tasks; doorknob and key tasks did not improve significantly.<br>• Muscle Strength (Force) - Grasp Force: Significantly greater increase after ReJoyce (4.1 N) compared to conventional therapy (1.5 N). Pinch Force: Improvements were not statistically significant for either group (ReJoyce: 1.2 N; Conventional: -0.3 N), likely due to emphasis on grasp-based games.<br>• Statistical Potency - ReJoyce ET: Large effect sizes (ARAT d=1.32; RAHFT d=1.95).Conventional ET: Small to moderate effect sizes (ARAT d=0.43; RAHFT d=0.32).<br>• Long-term Maintenance - Improvements were maintained for at least 3 months post-intervention. At the 30-week follow-up, scores remained significantly higher than baseline, with some groups even showing slight further increases.
Massey et al. (2025)	• iCycle FES-VR Training: 3 sessions per week for up to 12 weeks.<br>• Structure: Longitudinal extension of a previous 4-week pilot study<br>• Built-in checkpoints at 4 and 8 weeks allowed participants to conclude early or continue to 12 weeks<br>• IDT (VR): Cycling-racing game providing real-time biofeedback on voluntary power to promote active engagement. Integrated FES cycling.	• Walking Performance (Speed & Endurance) - 10MWT: Improved from baseline at 4 weeks (-10s for P1; -3s for P2). 6MWT: Improved from baseline at 4 weeks (+33m for P1; +135m for P2). Maintenance: Gains were maintained through the 12-week training period and the 2-month follow-up.<br>• Functional Independence - WISCI-II: Increased at 4 weeks (+3 for P1; +1 for P2) and maintained through the 2-month follow-up period.

	• Assessment: Comprehensive testing at Baseline, 4, 8, and 12 weeks, with follow-ups at 1 and 2 months post-training.	• Balance & Trunk Control - TIS: P1 showed an improvement of +4 points at 4 weeks. BBS: P1 showed incremental gains (Baseline: 30; 4-wks: 40; 8-wks: 39; 12-wks: 41).<br>• Biomechanical (Cycling Power) - Voluntary Power: Both participants showed a progressive increase in average voluntary cycling power across every 4-week training block (Weeks 4, 8, and 12).<br>• Qualitative (User Experience) - Participants reported that they enjoyed the VR racing game, stating it "distracted from their physical exertion" and provided high motivation to exercise.<br>• Neurological (ISNCSCI) - Motor: Both participants had high baseline motor scores (Grade 4 or 5). Sensory: No overall changes were observed in sensory scores.
Pizzolato et al. (2024)	• Digital Twin-Controlled BCI-FES-VR Cycling: ~150 min/week (2 sessions/week) for 52 weeks.<br>• IDT-Stim Interaction: Cycling intent detected via EEG (OpenBCI) processed by a Personalized Digital Twin (OpenSim/CEINMS physics-based model).<br>• Interface: Digital Twin predicted optimal muscle activations in real-time to adjust target cadence (0–30 rpm), FES intensity, and ergometer assistance/resistance.<br>• Stimulation parameters: Pulse width max 500 µs; current max 50 mA.<br>• VR: First-person avatar (Meta Quest 2) exploring natural environments.<br>• Adjunct: Buspirone hydrochloride (7.5 mg BID).	• Neurological (AIS Grade) - Conversion: After 12 months, 2/4 participants (P2 and P3) were reclassified from AIS A/B to AIS C.<br>• Sensory Function - All participants reported improved sensory function below the level of injury, including enhanced perception of touch, vibration, and temperature.<br>• Musculoskeletal (Bone) - Femoral Neck: Average BMD increase of 1.6% (individual range: -1.44% to +5.31%). Lumbar Spine: Average BMD increase of 4.2% (individual range: +0.26% to +8.04%).<br>• Musculoskeletal (Muscle) - Muscle Quality: All participants exhibited improved muscle quality characterized by a reduction in fat infiltration on MRI.<br>• Autonomic Function - Thermoregulation: Participant P4 self-reported improved thermoregulation, specifically recovering the ability to sweat and better manage hot weather.<br>• Feasibility - Setup Time: Average combined donning and doffing time was <15 minutes.
Sayenko et al. (2011)	• Videogame-based NMES ankle training: 3 sessions/week for 16 weeks (48 total sessions).<br>• Structure: 60 min sessions with 45 min of active NMES.<br>• Interface/Trigger: Participant used a joystick to manually modulate NMES intensity in real-time to navigate a virtual "snake".<br>• IDT (Visual Feedback): Target collection game ("Snake") on an LCD monitor; turning radius was controlled by the ankle joint angle detected by a tilt sensor.<br>• Stimulation parameters: 40 Hz frequency; 300s pulse duration; intensity ranged from 30–80 mA (PF) and 20–60 mA (DF).<br>• Resistance: Inverted pendulum (1m length, 3kg weight) provided isotonic and isometric resistance.	• Muscle Strength (Torque) - Plantarflexion: Significantly increased from 11.0 ± 1.7 Nm (1st session) to 27.0 ± 4.0 Nm (48th session). Dorsiflexion: Significantly increased from -5.1 ± 0.8 Nm (1st session) to -16.0 ± 2.0 Nm (48th session).<br>• Biomechanical (ROM) - Plantarflexion ROM: Increased from 17.7 ± 2.1° to 28.7 ± 3.4°. Dorsiflexion ROM: Increased from -2.9 ± 0.8° to -17.9 ± 1.5°.<br>• Muscle Endurance - Initially, NMES produced sufficient torque for only 20 minutes. By session 20, the participant could sustain effective muscle contractions for the full 45-minute training period.<br>• Performance - Game score improved dramatically from 12 points in the first session to 421 points by the 48th session.<br>• Fatigue Effects - During the 48th session, torque and ROM decreased toward the end (e.g., PF torque dropped to 11.1 Nm), yet these values remained significantly higher than the baseline measurements taken during the first session.<br>• Qualitative (Engagement) - The participant reported high motivation and enjoyment. He stated the game was challenging but the goal to maximize his score kept his attention focused throughout the sessions.

Sayenko et al. (2009)	• Videogame-based NMES ankle training: A single 45-minute session.<br>• Structure: Session involved approximately 300 repetitions of alternating plantarflexion (PF) and dorsiflexion (DF).<br>• Interface: Participant used an analogue joystick to manually control stimulation intensity in real-time; forward inclination triggered PF, while backward triggered DF.<br>• IDT (Visual Feedback): Target collection "Snake" game on a 37" LCD; joint angle determined the snake's turns (clockwise for PF, counter-clockwise for DF).<br>• Stimulation parameters: 40 Hz frequency; 300 μs pulse duration; intensity ranged from 20–30 mA (threshold) to 70–80 mA (80% of max torque).<br>• Resistance: Inverted pendulum (1m length, 3kg weight) provided resistance against movement.	• Muscle Strength (Torque) - Plantarflexion (PF): Peak torque remained stable, ranging from 15.3 ± 1.3 Nm to 16.4 ± 1.5 Nm. Dorsiflexion (DF): Peak torque remained stable, ranging from -9.2 ± 0.5 Nm to -9.9 ± 0.8 Nm.<br>• Biomechanical (ROM) - Plantarflexion ROM: Consistent across the session at approximately 19°. Dorsiflexion ROM: Ranged from -12.5 ± 0.4° to -9.4 ± 1.3°.<br>• Endurance & Fatigue - Repetitions: Approximately 300 repetitions of alternating PF and DF were completed. Maintenance: Performance and stimulation parameters (intensity/duration) remained stable throughout the 45-minute session with no signs of fatigue.<br>• Subjective (Psychological) - Engagement: Participant reported the game was "fun," "challenging," and "easy to control". Motivation: The goal of maximizing the score motivated the participant and maintained his attention for the entire session.<br>• Safety & Feasibility - Adverse Events: No safety concerns were noted; the participant was encouraged to continue the training on a regular basis. Adjustability: The system was successfully adjusted to meet the participant's specific needs.
Zoulias et al. (2019)	• FES Standing + Skiing Exergame: 2 sessions/week for 6 months (36–41 sessions).<br>• Pre-conditioning: 3 months of home-based FES knee extensions (60 min, 3–5x/week) until able to lift 1kg at the ankle.<br>• IDT (Exergame): Skiing simulator where the virtual skier's horizontal position is controlled by real-time bodyweight distribution.<br>• Stimulation parameters: 30 Hz frequency; pulse width 0–500 μs; amplitude 138 mA.<br>• Interface: Force plates, load cells, and 8-camera motion capture integrated via a Digital Twin-like data processing routine (Lab Streaming Layer).<br>• Logic: Cyclical posture-shifting (5s ON, 1s transition, 3s rest).	• Biomechanical (Lower Limb Loading) - Peak Loading: Achieved ground reaction forces (GRF) over 70% (and up to 80%) of full body weight (BW) on the supporting leg during each cycle. Efficiency: Participants supported almost all body weight through their legs, with arm support maintained at <10% BW (mean 7.59%).<br>• Longitudinal Improvements - Weight-Bearing Capacity: Average BW supported on the stimulated leg increased significantly by 13.88 percentage points, rising from 63.69% in early sessions to 78.61% in late sessions ($p = 0.0071$).<br>• Functional Endurance - Session Completion: All participants completed full 60-minute sessions starting from the second session. Repetitions: Participants reached 650–700 weight-shift cycles per session within the first 5–8 sessions and maintained this performance thereafter.<br>• Fatigue & Stability - Within-Session Stability: Leg support forces remained stable throughout the hour-long session with no discernible decline due to muscle fatigue.<br>• Postural Kinematics - Range of Motion (ROM): Trunk movement amplitude (measured by the clavicle marker) increased from 12.13° to 15.22°. While the trend was positive, this increase was not statistically significant in post-hoc testing ($p = 0.0966$).<br>• Qualitative (Engagement) - Motivation: The exergame provided an "interactive challenge" that motivated participants to actively try to increase the weight supported on their leg to achieve game goals (passing through virtual hoops).

Fig. S5. List of irretrievable records

1. #631

HEALTH CARE TECHNOLOGY TODAY.

[Unknown authors]

;7():

American Physical Therapy Association
